# Continued nintedanib in patients with systemic sclerosis-associated interstitial lung disease: 3-year data from SENSCIS-ON

**DOI:** 10.1136/rmdopen-2024-005086

**Published:** 2025-02-22

**Authors:** Yannick Allanore, Madelon C Vonk, Oliver Distler, Arata Azuma, Maureen D Mayes, Alexandra James, Veronika Kohlbrenner, Margarida Alves, Dinesh Khanna, Kristin B Highland, on behalf of the SENSCIS-ON trial investigators

**Affiliations:** 1 Department of Rheumatology A, Université Paris Cité, APHP, Cochin Hospital, Paris, France; 2 Department of Rheumatology, Radboud University Medical Center, Nijmegen, The Netherlands; 3 Department of Rheumatology, University Hospital Zurich, University of Zurich, Zürich, Switzerland; 4 Clinical Research Center, Mihara General Hospital, Saitama, Japan; 5 Nippon Medical School, Tokyo, Japan; 6 Division of Rheumatology and Clinical Immunogenetics, University of Texas McGovern Medical School, Houston, Texas, USA; 7 Elderbrook Solutions GmbH, Bietigheim-Bissingen, Germany; 8 Boehringer Ingelheim Pharmaceuticals Inc, Ridgefield, Connecticut, USA; 9 Boehringer Ingelheim International GmbH, Ingelheim am Rhein, Germany; 10 Scleroderma Program, Department of Medicine, University of Michigan, Ann Arbor, Michigan, USA; 11 Cleveland Clinic, Cleveland, Ohio, USA

**Keywords:** Connective Tissue Diseases, Pulmonary Fibrosis, Scleroderma, Systemic

## Abstract

**Objective:**

We assessed adverse events and changes in forced vital capacity (FVC) in patients treated with open-label nintedanib over 148 weeks of SENSCIS-ON, the extension of the SENSCIS trial.

**Methods:**

Adverse events and changes in FVC over 148 weeks of SENSCIS-ON were assessed in patients who received nintedanib in SENSCIS and continued nintedanib in SENSCIS-ON (‘continued nintedanib’ group) and in patients who received placebo in SENSCIS or received nintedanib for ≤28 days in a drug–drug interaction study and then received nintedanib in SENSCIS-ON (‘initiated nintedanib’ group).

**Results:**

The continued nintedanib group comprised 197 patients, and the initiated nintedanib group comprised 247 patients (231 from SENSCIS). Diarrhoea was the most frequent adverse event, reported in 152 (77.2%) and 183 (74.1%) patients in the continued nintedanib and initiated nintedanib groups, respectively. Among patients in the continued and initiated nintedanib groups, respectively, 53 (26.9%) and 148 (59.9%) had ≥1 dose reduction, 72 (36.5%) and 131 (53.0%) had ≥1 treatment interruption and 29 (14.7%) and 72 (29.1%) had adverse events that led to treatment discontinuation. Mean (SE) changes in FVC (mL) at week 148 were −189.1 (29.5) in the continued nintedanib group and −126.4 (26.4) in the initiated nintedanib group.

**Conclusion:**

The safety profile of nintedanib over 148 weeks of SENSCIS-ON was consistent with that reported in SENSCIS. Changes in FVC during SENSCIS and SENSCIS-ON supported a continued effect of nintedanib on slowing the decline in lung function, but showed continued progression of SSc-ILD.

**WHAT IS ALREADY KNOWN ON THIS TOPIC**

- The results of the randomised placebo-controlled SENSCIS trial showed that in patients with systemic sclerosis-associated interstitial lung disease (SSc-ILD), nintedanib reduced the rate of decline in forced vital capacity (FVC) over 52–100 weeks with an acceptable safety profile.

**WHAT THIS STUDY ADDS**

- The results of this open-label extension study show that the safety profile of nintedanib over longer-term use was consistent with that seen in the SENSCIS trial and that the changes in FVC over longer-term treatment support a continued effect of nintedanib on slowing the decline in lung function.

**HOW THIS STUDY MIGHT AFFECT RESEARCH, PRACTICE OR POLICY**

- These findings suggest that nintedanib can be used over the long term to slow the progression of SSc-ILD and so improve patient outcomes.

## Introduction

 Systemic sclerosis (SSc) is a complex autoimmune disease characterised by inflammation, vasculopathy and fibrosis of the skin and internal organs.^1^ Interstitial lung disease (ILD) is a frequent complication of SSc that may lead to pulmonary fibrosis.^2^ Nintedanib is a tyrosine kinase inhibitor with antifibrotic and anti-inflammatory effects.^3^ In the randomised placebo-controlled SENSCIS trial in patients with SSc-ILD, nintedanib reduced the rate of decline in forced vital capacity (FVC) over 52 weeks and over 100 weeks, with adverse events that were manageable for most patients.^4 5^ SENSCIS-ON was an open-label extension of the SENSCIS trial. Here, we report 3 year data from SENSCIS-ON.

## Methods

The designs of the SENSCIS and SENSCIS-ON trials (NCT02597933 and NCT03313180) have been described.^4 6^ Briefly, patients in the SENSCIS trial had SSc-ILD with the onset of first non-Raynaud symptom in the prior ≤7 years, the extent of fibrotic ILD on high-resolution computed tomography (HRCT) of ≥10%, FVC of ≥40% predicted and diffusion capacity of the lung for carbon monoxide 30–89% predicted. Patients receiving prednisone ≤10 mg/day or equivalent and/or stable therapy with mycophenolate or methotrexate for ≥6 months were allowed to participate. Patients who completed the SENSCIS trial on treatment (nintedanib or placebo) and attended a follow-up visit were eligible to enter SENSCIS-ON. Patients who completed an open-label drug–drug interaction study of nintedanib and Microgynon (ethinylestradiol and levonorgestrel), in which female patients with SSc-ILD received nintedanib over a period of ≥14 days to approximately 28 days,^7^ were also eligible to participate in SENSCIS-ON. In SENSCIS and SENSCIS-ON, to manage adverse events, the dose of nintedanib could be reduced from 150 mg twice daily to 100 mg twice daily, and the treatment could be interrupted (for ≤4 and ≤12 weeks in the case of events deemed related and unrelated to nintedanib, respectively); in addition, the investigators were provided with recommendations for the management of diarrhoea and liver enzyme elevations.^8^

We analysed changes in FVC and adverse events over 148 weeks of SENSCIS-ON in patients who had received nintedanib in the SENSCIS trial and continued to take it in SENSCIS-ON (‘continued nintedanib’ group) and in patients who had received placebo in the SENSCIS trial and initiated nintedanib in SENSCIS-ON or who had received nintedanib for a short period in the drug–drug interaction study (‘initiated nintedanib’ group). All analyses were descriptive and conducted in patients who received ≥1 dose of nintedanib in SENSCIS-ON. Adverse events were coded according to preferred terms in the Medical Dictionary for Regulatory Activities (MedDRA). Changes from baseline in FVC were based on observed data available at the respective time point. The SENSCIS-ON trial was carried out in compliance with the protocol and in accordance with the principles of the Declaration of Helsinki, the International Council for Harmonisation Harmonised Tripartite Guideline for Good Clinical Practice, applicable regulatory requirements and standard operating procedures. Patients provided written informed consent before entering the trial.

## Results

The continued nintedanib group comprised 197 patients, and the initiated nintedanib group comprised 247 patients (231 from SENSCIS, 16 from the drug–drug interaction study). Among the patients who participated in SENSCIS-ON, at the start of the parent trial, the mean (SD) age was 53.7 (11.9) years, time since the onset of the first non-Raynaud symptom was 3.6 (1.9) years and FVC was 73.4 (16.9)% predicted. The baseline characteristics of these patients at the start of SENSCIS-ON have been published.^6^ Briefly, in the overall population, the majority of patients were white (69.4%) and female (75.5%), with a mean (SD) age of 55.0 (11.9) years and mean (SD) FVC of 70.6 (18.0)% predicted. Some form of immunosuppression was taken by 236 patients (53.2%); mycophenolate was taken by 232 patients (52.3%).

In total, 126 (64.0%) and 125 (50.6%) patients in the continued nintedanib and initiated nintedanib groups, respectively, were receiving nintedanib at week 148 of SENSCIS-ON. The median (minimum, maximum) exposure to nintedanib in SENSCIS-ON was 35.8 (0.2, 35.8) months in the continued nintedanib group and 31.4 (0.0, 35.8) months in the initiated nintedanib group. The median (minimum, maximum) cumulative exposure to nintedanib across SENSCIS and SENSCIS-ON was 47.8 (12.8, 59.0) months.

Diarrhoea was the most frequent adverse event, reported in 152 patients (77.2%) who continued nintedanib and 183 patients (74.1%) who initiated nintedanib in SENSCIS-ON (table 1). The mean (SE) changes in weight from baseline to week 148 of SENSCIS-ON were −1.70 (0.51) kg in patients who continued nintedanib (n=117) and –3.75 (0.55) kg in patients who initiated nintedanib (n=118). Among patients who continued and initiated nintedanib in SENSCIS-ON, respectively, 53 (26.9%) and 148 (59.9%) had ≥1 dose reduction and 72 (36.5%) and 131 (53.0%) had ≥1 treatment interruption. Adverse events led to permanent discontinuation of nintedanib in 29 (14.7%) patients in the continued nintedanib group and 72 (29.1%) patients in the initiated nintedanib group (table 2). Adverse events led to permanent discontinuation of nintedanib in 22.0% and 23.6% of patients taking and not taking mycophenolate, respectively. Diarrhoea was the adverse event that most frequently led to permanent discontinuation of nintedanib (in 8 [4.1%] and 25 [10.1%] patients in the continued nintedanib and initiated groups, respectively). Serious adverse events were reported in 76 (38.6%) and 95 (38.5%) patients in the continued nintedanib and initiated nintedanib groups, respectively. Serious adverse events were reported in 38.8% and 38.2% of patients taking and not taking mycophenolate, respectively.

**Table 1:** Adverse events over 148 weeks of SENSCIS-ON

	Continued nintedanib(n=197)	Initiated nintedanib (n=247)
Diarrhoea	152 (77.2)	183 (74.1)
Nausea	43 (21.8)	73 (29.6)
Skin ulcer	48 (24.4)	54 (21.9)
Vomiting	38 (19.3)	59 (23.9)
Liver test abnormalities	31 (15.7)	63 (25.5)
Upper respiratory tract infection	39 (19.8)	33 (13.4)
Nasopharyngitis	31 (15.7)	40 (16.2)
Cough	36 (18.3)	33 (13.4)
Arthralgia	34 (17.3)	32 (13.0)
Weight decreased	23 (11.7)	33 (13.4)
Abdominal pain	10 (5.1)	36 (14.6)
Gastro-oesophageal reflux disease	24 (12.2)	15 (6.1)

Adverse events are shown based on single preferred terms, except for ‘liver test abnormalities’, which was based on the standardised MedDRA query ‘liver related investigations, signs and symptoms’ (broad definition). Data are n (%) of patients with ≥1 such event reported over 148 weeks (or until 7 days days after the last trial drug intake for patients who discontinued the trial drug before week 148). Events reported in >12% of patients in either group in SENSCIS-ON are shown.

**Table 2:** Adverse events that led to nintedanib discontinuation over 148 weeks of SENSCIS-ON

	Continued nintedanib(n=197)	Initiated nintedanib (n=247)
Diarrhoea	8 (4.1)	25 (10.1)
Alanine aminotransferase increased	0	7 (2.8)
Vomiting	0	6 (2.4)
Aspartate aminotransferase increased	0	5 (2.0)
Abdominal pain	0	5 (2.0)
Progression of ILD*	4 (2.0)	0

Adverse events are shown based on single MedDRA preferred terms. Data are n (%) of patients with ≥1 such event reported over 148 weeks. Events leading to discontinuation in ≥2% of patients in either group in SENSCIS-ON are shown.*Corresponds to the MedDRA preferred term .

* Corresponds to the MedDRA preferred term ‘interstitial lung disease’.

ILDinterstitial lung disease

**Figure 1:**
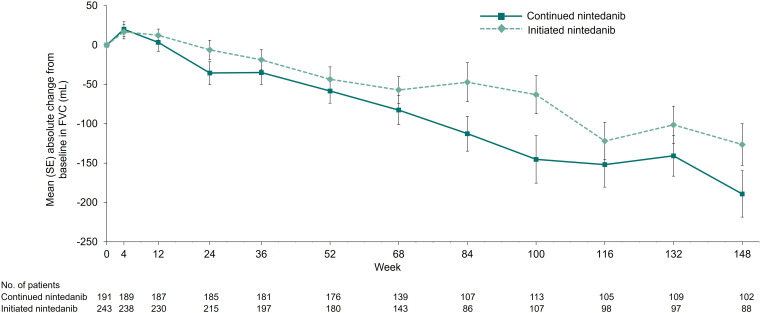
Change in FVC (mL) from baseline of SENSCIS-ON.

In total, 102 (51.8%) and 88 (35.6%) patients in the continued nintedanib and initiated nintedanib groups, respectively, had FVC data available at baseline and week 148. Mean (SE) changes in FVC from baseline to week 148 of SENSCIS-ON were −189.1 (29.5) mL in patients who continued nintedanib, −126.4 (26.4) mL in patients who initiated nintedanib and −160.0 (20.1) mL in all patients. Changes in FVC over 148 weeks in patients who continued and initiated nintedanib in SENSCIS-ON are shown in figure 1. Mean (SE) changes in FVC from baseline to week 148 of SENSCIS-ON were −170.5 (27.7) mL in patients treated with mycophenolate at baseline (n=115) and −144.0 (28.2) mL in patients not treated with mycophenolate at baseline (n=75).

## Discussion

These analyses of data from SENSCIS-ON extend the findings from the first 52 weeks of this trial^6^ and demonstrate that longer-term nintedanib treatment had an acceptable safety and tolerability profile, with no new safety signals observed. As observed in previous clinical trials of nintedanib in patients with various forms of pulmonary fibrosis^8^,^11^ and in studies based on data from clinical practice,^12^ diarrhoea was the most frequently reported adverse event. In SENSCIS-ON, diarrhoea was reported in approximately 75% of patients and, consistent with observations in the nintedanib group of the SENSCIS trial,^8^ 7.4% of patients (4.1% in the continued nintedanib group and 10.1% in the initiated nintedanib group) discontinued nintedanib due to diarrhoea. These findings reiterate the importance of educating the clinicians and patients who will be using nintedanib about how to manage diarrhoea if it occurs to help patients stay on treatment.^13^ Based on pooled data from clinical trials, among patients with ILDs other than systemic autoimmune rheumatic disease ILDs, the proportion of patients with adverse events leading to nintedanib discontinuation was greater in female than male patients.^14^ Data from real-world studies suggest that older age, female sex, lower body mass index, lower FVC % predicted and higher concomitant medication use may be associated with a higher risk of nintedanib discontinuation.^12 15^

Dose adjustments, interruptions and permanent discontinuations of nintedanib were more frequent among patients who initiated nintedanib than continued nintedanib in SENSCIS-ON. This was also observed in the data collected over the first 52 weeks of SENSCIS-ON.^6^ The rates of dose reductions, treatment interruptions and discontinuations of nintedanib over the first 52 weeks were similar in patients who initiated nintedanib in SENSCIS-ON as in patients who initiated nintedanib in the SENSCIS trial,^6 8^ supporting the need to manage adverse events in patients who are initiated on nintedanib. The observation of a lower rate of discontinuations in the patients who continued rather than initiated nintedanib in SENSCIS-ON may reflect selection bias in that the patients who continued nintedanib in the extension trial were more likely to be tolerating the drug.

The decline in FVC in patients with SSc-ILD is associated with an increased risk of hospitalisation and mortality.^2 16 17^ The decline in FVC observed during SENSCIS and SENSCIS-ON, despite the therapeutic effect of nintedanib, reflects the progressive nature of SSc-ILD in the population enrolled. However, SSc-ILD is known to have a heterogeneous and unpredictable clinical course,^16^ making it important that patients are closely monitored, including with regular pulmonary function tests.^18 19^

Nintedanib has been licensed for the treatment of SSc-ILD by regulatory authorities and received a conditional recommendation for use in the treatment of SSc-ILD in guidelines published by the American Thoracic Society^20^ and American College of Rheumatology.^21^ The declines in FVC over 52 weeks in the nintedanib group of SENSCIS and in SENSCIS-ON were similar (43 and 51 mL, respectively) and much smaller than the changes in FVC over 52 weeks in the placebo group of SENSCIS (105 mL). Over 148 weeks of SENSCIS-ON, the decline in FVC was 160 mL, suggesting a continued effect of nintedanib in slowing the disease progression, as also observed in patients with idiopathic pulmonary fibrosis.^11^

Strengths of our analyses include the standardised collection of FVC measurements, which was measured using sponsor-supplied spirometers and in accordance with American Thoracic Society/European Respiratory Society guidelines,^22^ and the duration of follow-up, which has provided the longest follow-up data on the safety and tolerability of nintedanib in patients with SSc-ILD. Limitations include the lack of a placebo group and the loss of patients throughout the trial. Comparisons between patients who continued and initiated nintedanib should be approached with caution.

In conclusion, the safety profile of nintedanib over 148 weeks of SENSCIS-ON was consistent with that reported in the SENSCIS trial, primarily characterised by gastrointestinal events that were manageable for most patients. Changes in FVC during SENSCIS and SENSCIS-ON supported a continued effect of nintedanib on slowing the decline in lung function in patients with SSc-ILD, but lung function continued to decline.
